# Aging-Induced Dysbiosis of Gut Microbiota as a Risk Factor for Increased *Listeria monocytogenes* Infection

**DOI:** 10.3389/fimmu.2021.672353

**Published:** 2021-04-28

**Authors:** Mohammad S. Alam, Jayanthi Gangiredla, Nur A. Hasan, Tammy Barnaba, Carmen Tartera

**Affiliations:** ^1^ Center for Food Safety and Applied Nutrition, U.S. Food and Drug Administration, Laurel, MD, United States; ^2^ CosmosID, Rockville, MD, United States; ^3^ Center for Bioinformatics and Computational Biology (CBCB), University of Maryland, College Park, MD, United States

**Keywords:** *Listeria monocytogenes*, listeriosis, aging, dysbiosis, inflammation, gut microbiota, metagenomics

## Abstract

Invasive foodborne *Listeria monocytogenes* infection causes gastroenteritis, septicemia, meningitis, and chorioamnionitis, and is associated with high case-fatality rates in the elderly. It is unclear how aging alters gut microbiota, increases risk of listeriosis, and causes dysbiosis post-infection. We used a geriatric murine model of listeriosis as human surrogate of listeriosis for aging individuals to study the effect of aging and *L. monocytogenes* infection. Aging and listeriosis-induced perturbation of gut microbiota and disease severity were compared between young-adult and old mice. Young-adult and old mice were dosed intragastrically with *L. monocytogenes*. Fecal pellets were collected pre- and post-infection for microbiome analysis. Infected old mice had higher *Listeria* colonization in liver, spleen, and feces. Metagenomics analyses of fecal DNA-sequences showed increase in α-diversity as mice aged, and infection reduced its diversity. The relative abundance of major bacterial phylum like, *Bacteroidetes* and *Firmicutes* remained stable over aging or infection, while the *Verrucomicrobia* phylum was significantly reduced only in infected old mice. Old mice showed a marked reduction in *Clostridaiceae* and *Lactobacillaceae* bacteria even before infection when compared to uninfected young-adult mice. *L. monocytogenes* infection increased the abundance of *Porphyromonadaceae* and *Prevotellaceae* in young-adult mice, while members of the *Ruminococcaceae* and *Lachnospiraceae* family were significantly increased in old mice. The abundance of the genera *Blautia* and *Alistipes* were significantly reduced post-infection in young-adult and in old mice as compared to their uninfected counterparts. Butyrate producing, immune-modulating bacterial species, like *Pseudoflavonifractor* and *Faecalibacterium* were significantly increased only in old infected mice, correlating with increased intestinal inflammatory mRNA up-regulation from old mice tissue. Histologic analyses of gastric tissues showed extensive lesions in the *Listeria*-infected old mice, more so in the non-glandular region and fundus than in the pylorus. Commensal species like *Lactobacillus*, *Clostridiales*, and *Akkermansia* were only abundant in infected young-adult mice but their abundance diminished in the infected old mice. Listeriosis in old mice enhances the abundance of butyrate-producing inflammatory members of the *Ruminococcaceae*/*Lachnospiraceae* bacteria while reducing/eliminating beneficial commensals in the gut. Results of this study indicate that, aging may affect the composition of gut microbiota and increase the risk of invasive *L. monocytogenes* infection.

## Introduction

*Listeria monocytogenes* is a Gram-positive, aerobic/facultative-anaerobic intracellular bacterium that can infect both humans and animals, including livestock, after ingestion of contaminated food. Human listeriosis caused by *L. monocytogenes* can manifest on a variety of syndromes including gastroenteritis, septicemia, meningitis and chorioamnionitis, and is associated with a high mortality rate (20%-30%). Invasive listeriosis is a more severe form of disease and affects certain high-risk groups of the population such as pregnant women and their fetuses, patients undergoing treatment for cancer, AIDS, organ transplant recipients, infants, and the elderly.

The incidence of listeriosis and the relative risk of infection vary significantly among population subgroups (1). Recently Pohl et al. (2) estimated that in the US, the annual incidence of listeriosis for adults ≥70 years was 1.33 cases per 100,000, while the incidence among the general population was 0.28 cases. In Europe, listeriosis incidence has increased among males ≥ 75 years, and females ≥25 years (2). CDC surveillance data suggest that the number of invasive listeriosis cases increases as people age, and for people ≥50 years of age, that increase is doubled for each 10-year increase in age (3). In general, aging is associated with a higher degree of morbidity in elderly populations due to life-stage-dependent changes in host-immune responses and decreasing ability to fight off systemic infections, like *Listeria* infection. Animal model studies have suggested that age-dependent dysregulation of innate immunity can impair adaptive immune responses and, as a result, altered T-helper effector cells cannot maintain a sustained CD8^+^ T cell cytokine response required for clearance of foodborne pathogens (4, 5). In addition, recent *L. monocytogenes* animal model studies using intragastric inoculation with Lmo-*InlA* ^m^ strain suggest that dysregulation of the Th1/Th2 response in aging mice may contribute to higher susceptibility to infection (6, 7).

It has recently become evident that gut microbiota has an important role on the host immune system, metabolism, and even behavior of the host (8, 9). Shifts or imbalances in the composition of gut microbiota has been correlated with many immunological, metabolic, and mental disorders (10). Previous research studies demonstrated that changes associated with aging are recognized by the indigenous microbiota that co-evolved together with its host as a part of the holobiont (11, 12). Moreover, commensal intestinal microbiota (or gut microbiota) confers natural resistance (colonization resistance) against orally acquired bacterial pathogens (13). Intestinal microbiota is believed to directly suppress invading pathogens by producing bacteriocins, by competing for nutrients, and indirectly by modulating host defense pathways. Studies (14–16) have shown that structural changes can happen in the gut that result in dysbiosis: a decrease in the number and diversity of beneficial bacteria (e.g. *Bifidobacteria*) and a corresponding increase in the number and diversity of harmful bacteria (e.g. *Clostridia*, *Enterobacteria*). In general, dysbiosis is any change to the composition of resident commensal communities relative to the community found in disease free state which can be characterized by: loss of microbial diversity, change in composition, including blooms of pathobiont and decrease in commensal or potentially beneficial bacteria (15, 16).

The gut microbiota is a very complex and diverse community of commensal bacteria that intimately interacts with the epithelium and underlying mucosal immune cells in the gastro-intestinal tract (17). A recent study by Becattini et al. reported that commensal microbes like the *Clostridiales* act as first line of defense against *L. monocytogenes* infection in mice (18). So, changes in the populations of commensals may increase the susceptibility of the elderly to foodborne infection. Aging is a recognized risk factor for increased susceptibility to listeriosis in humans and mice (2, 5–7), but little is known about the risk of listeriosis as a function of altered gut microbiota due to aging. In other at-risk populations for listeriosis, such as infants or pregnant women, intestinal dysbiosis has been implicated as a factor associated with susceptibility due to altered microbial profiles with marked reduction in *Clostridiales* members and increase in *Proteobacteria* (18, 19).

Our previous studies using a geriatric listeriosis murine model, suggested increased susceptibility to oral *L. monocytogenes* infection in the old mice due to an imbalance of pro- and anti-inflammatory responses (6, 7). We also observed increased Th1/Th2 responses in the old mice that were aged normally. We hypothesize that susceptibility in mice and humans to *L. monocytogenes* infection might depend on the diversity of gut microbiota that can modulate immune response in the gut. Thus, aging-mediated perturbations/shifts of microbiota would further increase the risk of *L. monocytogenes* infection. In the present study, we used a geriatric murine model of listeriosis as a human surrogate of listeriosis for elderly persons to study aging-induced alteration of commensal microbiota as a risk of invasive *L. monocytogenes* infection. Listeriosis-induced perturbation of gut microbiota and disease severity were also compared between young-adult and old mice and correlated with intestinal pathologies.

## Materials and Methods

### Mice

Four-week old female C57BL/6 mice were purchased from The Jackson Laboratory (Bar Harbor, ME) and allowed to age in-house under specific pathogen free (SPF) conditions until use. All mice were categorized as young-adult (2-months) or old (20-months). Aging mice were regularly monitored for senescent changes and only healthy mice were used in the experiments.

### Ethical Statements

All experiments were conducted in accordance with the recommendations in the Guide for the Care and Use of Laboratory Animals of the National Research Council. The protocol (protocol approval number: BFQ-11-006) was approved by the Food and Drug Administration, Center for Food Safety and Applied Nutrition-Institutional Animal Care and Use Committee (CFSAN-IACUC). All mice were kept at the MOD-1, CFSAN/FDA, AAALAC (American Association for Accreditation of Laboratory Animal Care) accredited animal facility. Approved standard animal husbandry protocol was followed for the care of mice.

### *Listeria monocytogenes* Infection of Mice

*L. monocytogenes* was grown on BHI agar plates (BBL, Becton and Dickinson, MD) at 37°C for 18 h. Young-adult (n=5–6) and old mice (n=5–6) were gavaged for two consecutive days (day zero and day-1) with *L. monocytogenes* Lmo-*InlA* ^m^ (gift from Dr. Wolf-Dieter Schubert & Dr. Thomas Wollert, HZI, Germany) at a dose of 1 × 10^6^ CFU in 100 μl of PBS. For each age group, control (young-adult control and old control) mice (n=5–6) were gavaged with 100 μl of PBS. The Lmo-*InlA* ^m^ strain is a murinized *L. monocytogenes* strain that is capable of invasion of mouse intestinal tissue and results in systemic infection. The Lmo-*InlA* ^m^ strain was modified from the wild-type Lmo-EGD strain by exclusively replacing the gene inlA with inlA^S192N-Y369S^ to produce the mutant strain Lmo-*InlA* ^m^ (20).

Mice were fasted for 4-6 hours prior to infection. Metal wire-flooring was used to prevent coprophagia. Mice were monitored daily, for 7 days, for clinical signs of disease. Infected mice that became severely sick were euthanized as per FDA-CFSAN-IACUC guidelines. All mice were also weighed and euthanized on day seven of infection. Spleen and liver tissues were collected for enumeration of *L. monocytogenes* colonization by viable colony counts. Fresh feces from the mice were collected in dry-ice and kept frozen at -80°C until use.

### Histopathology, Immunostaining, and Intestinal Damage Scoring

Portions of liver tissues were fixed in Bouin’s solution (Ricca Chemical, Arlington, TX), washed with ethanol, embedded in paraffin, cut into 3-5-*μ*m sections, and stained with hematoxylin and eosin (H&E). For gastric tissue analyses and histological evaluation, longitudinal segments, including the antrum and corpus plus proximal duodenum, were fixed in Bouin’s fixative solution (Ricca Chemical) for 24 h, washed twice with 70% ethanol and embedded in paraffin, cut into 3- to 5-μm sections, and stained with hematoxylin and eosin. For immunohistochemistry, similar 3-μm gastric sections were stained with polyclonal anti-MPO (Myeloperoxidase) antibody (Novus Biochemicals, Littleton, CO), and tissue-bound peroxidase activity was visualized with DAB (3, 3′-diaminobenzidine). Hematoxylin was used for nuclear counter staining. MPO-positive cells were shown in the tissue with an arrow. All slides were scanned and digitally stored using a Nanozoomer (Hamamatsu, Japan) with NDP-view-2 software (Hamamatsu). Scanned H&E images were used for measuring tissue area or height at suitable magnification with NDP-view-2 software. Gastric inflammation was assessed using a modified scoring system, as previously described (21). Briefly, two sections were collected from each stomach, and each region of the stomach (forestomach or cardia, corpus, and antrum) was assessed individually for three parameters; (1) thickening, (2) infiltration of polymorphonuclear cells and (3) infiltration of MNCs (mononuclear cells). Severity was graded based on the absence (0) or presence (1) of each parameter, with polymorphonuclear infiltration further examined (absence or presence) for focal, diffuse, or abscess involvement. Similarly, MNC infiltration was examined for focal, diffuse, or aggregate involvement in the lamina propria. A total score was calculated by summing the score values for each region of the stomach for one section. Results are reported as total damage scores.

### Enumeration of *Listeria monocytogenes* Colonization in Liver and Spleen Tissues

For the measurement of *L. monocytogenes* burden, portions of liver and spleen tissue were homogenized in PBS, and replicate serial 10-fold dilutions were plated onto BHI agar plates and incubated overnight at 37° C. Bacterial counts were determined by viable colony count method.

### RNA Extraction and Real-Time RT-PCR

Total RNA was extracted from infected and uninfected tissues using Qiagen RNA extraction kits (Qiagen, Valencia, CA). In each case, RNA was reverse-transcribed to yield cDNA using the RT^2^ First Strand kit (Qiagen). Transcripts were measured by Real-time RT-PCR with a CFX96 Real-Time System (BioRad, Irvine, CA) using RT^2^ SYBR Green qPCR and RT^2^ qPCR primers (IFN-γ: PPM03121A, IL-10: PPM03017B, and IL-17:Add IL-17: PMM03023A, IL-23: PMM03763F) from Qiagen-SABiosciences (Frederick, MD). The levels of RNA for the target sequences were determined by melting curve analysis using the Bio-Rad CFX manager software as previously described (7). Normalized levels of each mRNA were determined using the formula 2^(Rt – Et)^, where Rt is the threshold cycle for the reference gene (GAPDH: PPM02946E, Qiagen) and Et is the threshold cycle for the experimental gene (ΔΔC_T_ method). Data are expressed as arbitrary units.

### Mouse Fecal DNA Extraction

Fecal DNA was extracted from infected and uninfected mouse feces using QIAamp DNA mini kits (Qiagen, Valencia, CA). Extracted DNA samples were kept at – 20°C.

### DNA Sequencing

DNA sequencing libraries were prepared with the Nextera XT DNA library preparation kit and Nextera indices (Illumina, San Diego, CA). Libraries were sequenced on a MiSeq platform using a MiSeq 500 cycle version 2 reagent kit (Illumina).

### Metagenomic Analyses

Unassembled metagenomic sequencing reads were analyzed using the CosmosID Metagenomics Cloud Application as previously described (22–25) to achieve multi-kingdom microbiome analyses and quantification of organisms’ relative abundance. This is defined as the proportion of unique organism-specific k-mers annotated by each database relative to the total number of unique sequencing reads generated for that sample. Briefly, the application utilizes GenBook^®^, a series of proprietary databases curated extensively by CosmosID Inc. (CosmosID Inc., Rockville, MD, USA), which is composed of over 150,000 microbial genomes and gene sequences representing over 15,000 bacterial, 5,000 viral, 250 protozoan, and 1,500 fungal species, as well as over 5,500 antibiotic resistance and virulence-associated genes. Metagenomic analyses for microbial composition levels based on changes that occurred in the gut microbiome due to *L. monocytogenes* infection were further analyzed using our in-house k-mer database (k=30) (26) for taxonomical identification of microbes to the species level; the total relative abundance of each organism in each sample was determined.

### Statistical Analysis

Statistical models for estimating microbial diversity and microbial community comparison methodology and metrics were performed using the STAMP (Statistical Analysis of Metagenomic Profiles) software package (27). The effect sizes and confidence intervals in microbial composition shifts were calculated between groups (uninfected control *vs Listeria*-infected relative to both young-adult and old mice). Gastric damage scoring and mRNA results are expressed as mean ± SEM or mean ± SD. Data were compared by Student’s *t* test (unpaired) or ANOVA, and results were considered significant if *p* values were less than 0.05. At least two independent experiments were performed.

## Results

### Fecal Microbiota Population Diversity Differs Between Young-Adult and Old Mice

**Figure 1:**
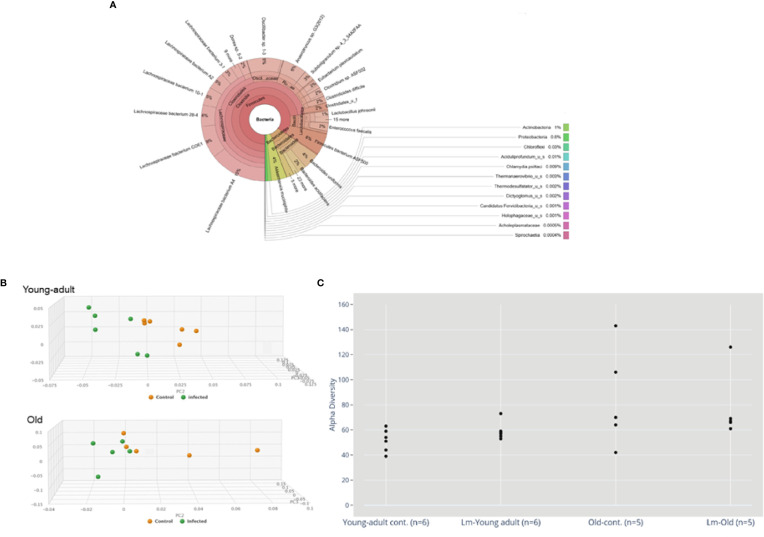
**(A)** Krona visualization of all bacteria detected across all mice tested. **(B)** Principal component analysis (PCA) of the fecal microbiota from young-adult and old C57BL/6 mice infected with *Listeria monocytogenes* (Lm) after 7-days post infection. The clustering on two PCA plots show control (uninfected) versus *L. monocytogenes* infected mice. Each symbol represents one mouse. Principal component analysis scores are plotted based on the relative abundance of total microbiota. Proportion of variance in each principal coordinate axis is denoted in the corresponding axis label. The uninfected control (brown circle) and infected mice (green circle) show clear separation. **(C)** Alpha diversity comparisons based on CHAO1. *Listeria* infection in old mice significantly lowers microbial richness compared with the uninfected old control mice.

Sequence analyses of DNA extracted from the samples revealed a wide diversity of bacteria, representing over 250 species, 125 genera and 15 bacterial phyla. **Figure 1A** depicts a Krona (28) visualization of all bacteria detected across all mice tested. The predominant phylum of bacteria were the gram-positive *Firmicutes*, *Bacteroidetes* and *Verrucomicrobia* representing 85%, 10% and 4% of total bacterial diversity, respectively. The phylum *Proteobacteria*, which includes a wide variety of pathogens, and *Actinobacteria*, which includes a wide variety of pathogens as well as symbionts, represented only 0.6% and 1% of total bacterial diversity.

We also evaluated whether gut microbiota differed between young-adult (2-months) and old (20-months) female mice. As depicted in **Figure 1B**, the principal component analysis (PCA) of gut microbiota based on their taxonomic abundance, both uninfected (control) young-adult and old mice clustered separately from *L. monocytogenes* infected young-adult and old mice. **Figure 1C** represents the alpha-diversity that evaluated distinguishable richness in taxa that were detected in fecal samples from both young-adult and old mice before and after *L. monocytogenes* infection. All old mice showed an increased alpha-diversity based on species richness (e.g. CHAO1 index (29) as compared to uninfected young-adult mice, and *L. monocytogenes* infection altered that diversity.

**Figure 2:**
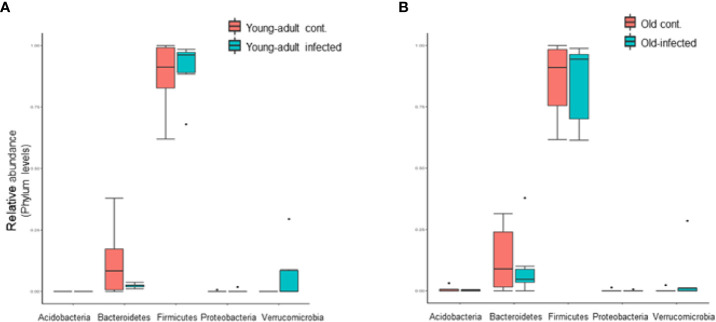
*Listeria monocytogenes* infection induced changes in the relative abundance of fecal microbiota at the phylum level. **(A)** Relative abundance of major bacterial Phylum in young-adult control and *L. monocytogenes*-infected young-adult mice. **(B)** Relative abundance of Phylum bacteria in old control and old *L. monocytogenes* infected mice. Data from mean ± SEM from a representative experiment using 4–6 mice. *p < 0.05.

We further analyzed the relative abundance of five major phyla of bacteria, which include *Firmicutes*, *Bacteroidetes*, *Verrucomicrobia*, *Actinobacteria*, and *Proteobacteria*, in mice fecal microbiota before and after infection. As shown in **Figure 2**, the majority of the fecal microbiota (~90%) are represented from the phyla *Bacteroidetes* and *Firmicutes*, with the remaining phyla combined, representing less than 7% of the total bacteria. We did not observe any changes in the combined abundance of *Bacteroidetes* and *Firmicutes* phyla with respect to aging in these mice; however, an interplay between increased abundance of *Firmicutes* followed by reduced abundance of *Bacteroidetes* was observed among mice infected with *L. monocytogenes*. This observation is supported by previous studies (9, 30) which suggested an increased *Firmicutes* to *Bacteroidetes* ratio in old mice as compared to young-adult mice. Interestingly, we saw a significant shift in the abundance of *Verucomicrobia* in both young-adult and old mice after *L. monocytogenes* infection, but their abundance was distinctly more in *Listeria*-infected young-adult mice when compared with old-infected mice.

### *Listeria monocytogenes* Infection Alters Microbiota Population Diversity in Young-Adult and Old Mice

**Figure 3:**
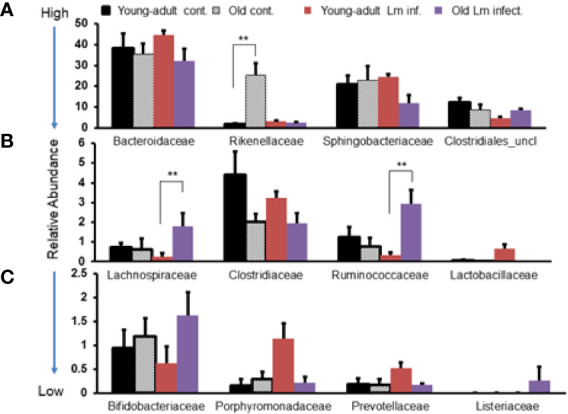
Comparative representation of relative abundance of fecal microbiota at the family level in young-adult and old mice before and after *Listeria monocytogenes* infection. **(A)** Showing high abundance bacteria (>10%) at the family level. **(B)** Intermediate abundance level (<6%) bacteria at the family level. **(C)** Low level (<2%) bacteria at the family level. Taxonomic composition for the abundant bacteria at the family level was generated from k-mer analyses based on taxonomic profiling. Data from mean ± SEM from a representative experiment using 4–6 mice. **p < 0.01.

In addition, we evaluated the taxonomic differences of fecal microbiota at the family level and compared the distribution based on high-to-low abundances across two age groups and infection status as shown in **Figure 3**. We selected the twelve most abundant families based on k-mer database metagenomics analyses and determined if particular families of bacteria are associated with aging and/or if *Listeria* infection perturbed abundance. *Bacteroidaceae*, *Sphingobacteriaceae* and *Clostridiales_uncl* bacteria all together comprised nearly 80% of the total fecal bacteria in the gut and was not significantly perturbed due to aging. Interestingly only the *Rikenellacae* family was most significantly (p<0.05) over abundant within old mice as compared with young-adult mice before infection; their abundance was significantly (p<0.05) reduced when old mice were infected. We do not know the reason for such reduction after infection in the old mice. We also saw marked reduction in the order *Clostridiales*, and the *Clostridiaceae* family as the mice aged. The members of the *Lactobaccillaceae* family bacteria, known for bacteriocins production and their protective antimicrobial roles (31–33), were found in low- abundance in old mice as compared to uninfected young-adult mice. Interestingly, *L. monocytogenes* infection significantly (p<0.05) increased their abundance only in young-adult mice. We observed marked increases in the abundance of *Porphyromonadaceae* and *Prevotellaceae* family bacteria only in the infected young-adult mice. On the other hand, both *Lachinospiraceae* and *Ruminococcaceae* increased significantly (p<0.05) in *Listeria*-infected old mice. We are unsure of the reason that *L. monocytogenes* infection caused a differential increase in the relative abundances in young-adult mice compared to old mice. We also detected an abundance of the *Listeriaceae* family bacteria in feces from infected old mice (**Figure 3C**).

### Aging Reduces Specific Genus Level Commensal Bacteria Responsible for Colonization Resistance Against *Listeria monocytogenes* Infection in Mice

**Figure 4:**
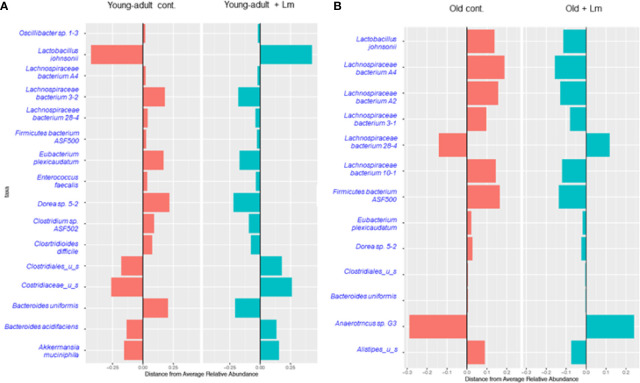
Centroid classification based on relative abundance of fecal microbiota at the genus and species level in young-adult and old mice after *Listeria monocytogenes* (Lm) infection. **(A)** Top sixteen bacterial species that showed either an increase or decrease abundance in young-adult mice before and after *L. monocytogenes* infection. **(B)** Top thirteen frequently bacterial species that showed either an increase or decrease abundance in old mice before and after *L. monocytogenes* infection.

We performed centroid classification of fecal microbiota at the genus and species level based on their relative abundance in young-adult and old mice after *L. monocytogenes* infection (**Figure 4**), and identified several commensal bacteria that are often reported to have significant protective roles during infection and/or disease. We detected sixteen species of bacteria in young-adult mice and thirteen species in old mice, with eight species common in between both age groups and their abundances altered after infection. Besides *Lachnopiraceae* family bacteria, which are less abundant in young-adult mice, seven additional bacterial species (*Oscillibacter* sp. 1-3, *Enterococcus faecalis*, *Clostridium* sp. ASF502, *Clostridioides difficile*, *Clostridaceaea*_u_s, *Bacteroides uniformis* and *Akkermansia muciniphila*) were only abundant in the feces of the young-adult mice. On the other hand, *Anearotruncus* sp. (G32012) and *Alistipes*_u_s bacteria were only detected in old mice. *Akkermansia muciniphila* (Phylum *Verrucomicrobia*), a mucin degrading bacterium often associated with a healthy gut were only present in the young-adult mice and barely detected in any of the old mice. Fransel et al. (9) also reported a similar increased abundance of *Akkermansia* in young-adult mice. *Clostridium* sp. ASF 502, *Clostridioides difficili*, *Clostridiales* u_s and *Clostridiaceae* u_s belonging to *Clostridiaceae* and *Clostridiales* groups were only abundant in young-adult mice but not in old-adult mice.

Recently, Becattini et al. (18) showed that several bacterial species including, majority of taxa belonging to the order *Clostridiales*, are associated with protection against *in vivo L. monocytogenes* infection. Interestingly, this group of bacteria are mostly absent in the feces of old mice suggesting that the increased susceptibility to *L. monocytogenes* infection in these old mice may be due to decreased colonization resistance. We also observed a decreased abundance of *Lactobacillus johnsonii* in the old mice as compared to young-adult mice. Commensals such as *Lactobacilli* spp., are previously known to produce anti-listerial bacteriocins in *in vivo* experiments and were shown to increase resistance to *Listeria* infection in mice with an intact microbiota (31). In general, both species-diversity and abundance were relatively reduced in old mice when compared to young-adult mice suggesting a decreased microbiota diversity or dysbiosis as mice ages.

We also observed an increase in relative abundance and emergence of diverse species of the *Lachnospiraceae* family bacteria, including *Dorea* sp. mostly present in old mice as compared to young-adult mice (**Figure 4**). A least three new *Lachnospiraceae* species (*Lachnospiraceae* bacterium A2*, Lachnospiraceae* bacterium 3-1 and *Lachnospiraceae* bacterium 10-1) were detected in old mice.

**Figure 5:**
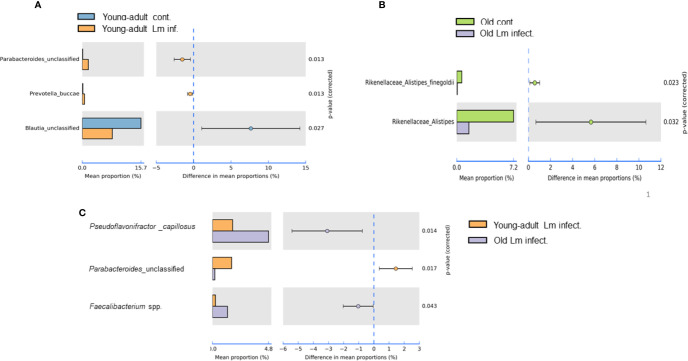
Species level relative abundance of fecal bacteria in young-adult and old mice before and after *Listeria monocytogenes* infection. **(A)** *Listeria*-infection induced changes of bacteria *Parabacteroides*, *Prevotella buccae*, *Blauia* in young-adult mice before and after *Listeria monocytogenes* infection. **(B)** *Listeria*-infection induced changes of bacterial species of the *Rikenellaceae* family that included the genus *Alistipes*, in particular *Alistipes finegoldii*, in old mice before and after *Listeria monocytogenes* infection. **(C)** *L. monocytogenes* infection induced changes in abundance *Pseudoflavonifractor capillosus*, *Parabacteroides*, and *Faecalibacterium* spp. in young-adult and old mice. Relative abundance levels were calculated at the 95% confidence intervals and *p*-value calculated for significance as shown in the figure. The relative abundance of Taxa at the genus level were generated from a k-mer database used for taxonomic profiling. Data from mean ± SEM from a representative experiment using 4–6 mice.

Our metagenomic analyses further suggested significant presence of *Parabacteroides_unclassified*, *Prevotella buccae* and *Blautia_unclassified* bacteria in young-adult mice (**Figure 5A** **)**. Both *Parabacteroides_unclassified* and *Prevotella buccae* which belong to *Porphyromonadaceae* and *Prevotellaceae* family respectively, were significantly (*p*=0.013) increased after infection in young-adult mice On the other hand, *Blautia_unclassified*, which belongs to *Lachnospiraceae* family were significantly (p=0.027) decreased in infected young-adult mice as compared to uninfected mice. It is important to note that, *Blautia* was not detected in old mice. *Alistipes finegoldii*, a commensal bacterium belonging to *Rikenellacdeae* family are exclusively present in the old mice We observed, *Listeria* infection significantly reduced its abundance in old mice (**Figure 5B**).

*Pseudoflavonifractor capillosus*, *Feacalibacterium* spp. and *Anaerotruncus* sp. G3 (2012) bacteria, all butyrate producers, were identified as highly abundant in infected old mice (**Figures 4B**, **5C**) but the abundance of *Parabacteroides*_unclassified decreased in infected old mice (**Figure 5C**). *Feacalibacterium* spp. and *Anaerotruncus* sp. G3 (2012) species belong to *Ruminococcaceae* family bacteria. As shown before we observed a significant (p<0.05) rise in abundance of *Ruminococcaceae* family bacteria only in the *L. monocytogenes*-infected old mice as compared to *L. monocytogenes*-infected young-adult mice (**Figure 3B**). Our analysis of the metagenomic data associated with bacteria belonging to the *Listeriaceae* family, we were able to detect the *L. monocytogenes* with which our experimental mice were orally infected, confirming persistence of *L. monocytogenes* in the feces on day seven of infection. Fecal samples from only infected-old mice showed higher abundances for *L. monocytogenes* strain (in 40% of the infected old mice with ~100% match to *EGD-e*) (data not shown). We were unable to detect the *InlA^m^* mutation from these samples in our read mapping results, due to the low abundance of reads associated with *Listeria* identified from these samples.

### Increased Intestinal Tissue Pathology and Inflammatory Immune-Biomarker Response in the Old Mice

**Figure 6:**
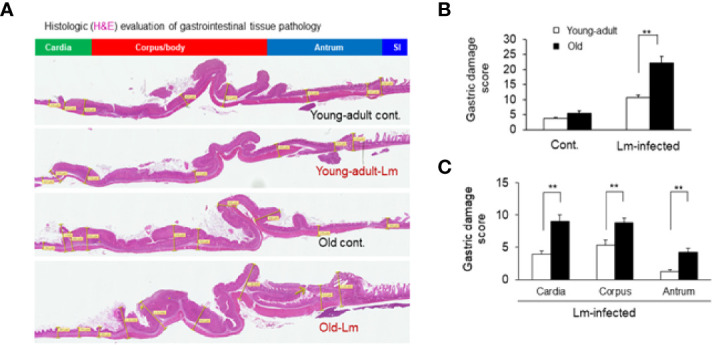
Gastric inflammation was more severe in *Listeria monocytogenes*–infected old mice. Mice were infected by gavage with 1 x 10^6^ CFU of *L. monocytogenes* per inoculation for two days, in consecutive, separate inoculations. Mice were euthanized 7-days post infection, and gastric tissue was processed for histologic examination. **(A)** Hematoxylin-eosin–stain of gastric sections from representative control (uninfected) or infected young-adult mice (top two), and control (uninfected) or infected old mice (bottom two). **(B, C)** Histologic scoring of gastritis in uninfected and infected young-adult and old mice. Data from mean ± SEM from a representative experiment using 4–6 mice. **p < 0.01.

**Figure 7:**
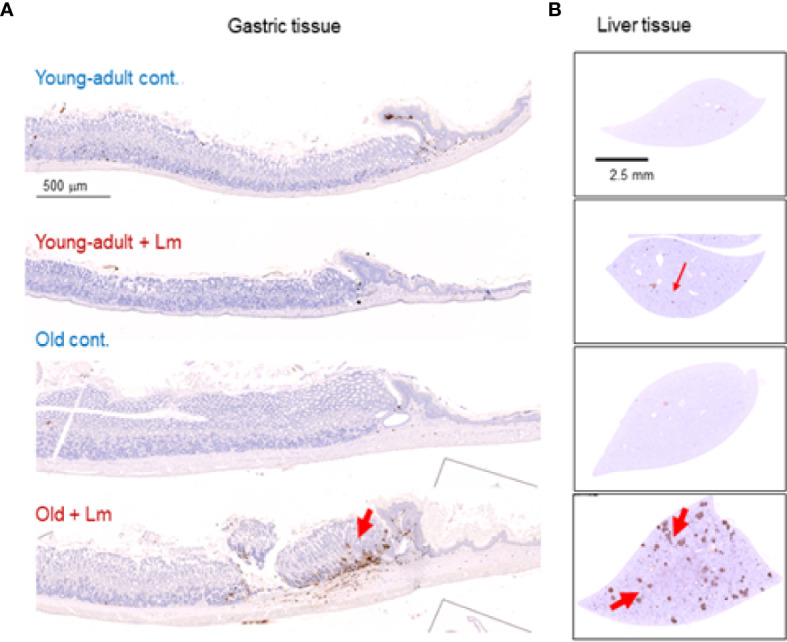
Increased gastritis and liver inflammation in old mice as compared to young-adult mice after oral *Listeria monocytogenes* infection analyzed by immunohistochemical evaluation of gastrointestinal tissue by myeloperoxidase (MPO) staining (neutrophils). **(A)** Myeloperoxidase (MPO) (left column) gastric sections from representative control (uninfected) or infected young-adult mice (top two), and control (uninfected) or infected old mice (bottom two). Arrows denote cells expressing MPO. Only a few scattered mononuclear cell (MNCs) and MPO-positive granulocytes can be seen in the submucosa and lamina propria, with no abnormal thickening of the gastric wall noted in uninfected control mice. Severe gastritis with dense MNC infiltration and defused MPO-positive granulocytes were noted in the submucosa and mucosa of the old mice in the cardia region. MNC aggregates between the glands spanned the entire width of the mucosa. **(B)** Myeloperoxidase (MPO) (right column) liver sections from representative control (uninfected) or infected young-adult mice (top two), and control (uninfected) or infected old mice (bottom two). Arrows denote cells expressing MPO. Data from mean ± SEM from a representative experiment using 4–6 mice.

**Figure 8:**
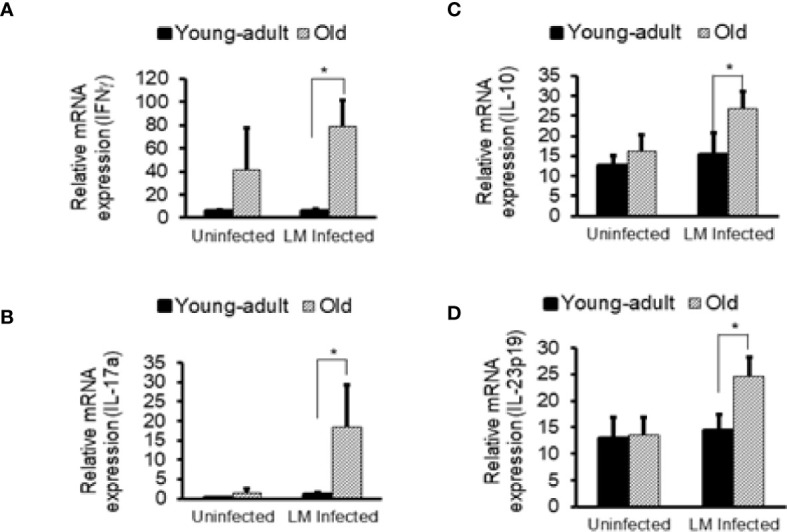
Increased *in vivo* inflammatory cytokine mRNA responses in the intestinal tissue from *Listeria monocytogenes*-infected old mice. Intestinal tissues from uninfected and *L. monocytogenes* (Lm)-infected mice of both age groups at 7-days post infection were used for RNA extraction and IFN-γ **(A)**, IL17a **(B)**, IL-10 **(C)**, and IL-23 **(D)** mRNA expression were measured. Data from mean ± SEM from a representative experiment using 4–6 mice. *p < 0.05.

Previously, we showed that old C57BL/6 mice were more susceptible to infection and had significant inflammatory changes in liver and spleen tissues after repeated gavage with *L. monocytogenes* (7). This time, we evaluated gastrointestinal pathology and inflammatory response after *L. monocytogenes* infection. Histologic scoring of gastritis in uninfected and infected young-adult and old mice are shown in **Figure 6**. We found infected older mice had significantly (p<0.05) more gastric pathology (**Figures 6B, C**). **Figure 7** shows the gastritis and liver inflammation in old mice as compared to young-adult mice after oral *L. monocytogenes* infection, as analyzed by immunohistochemical evaluation of tissue by myeloperoxidase (MPO) staining (neutrophils). Severe gastritis with dense mononuclear cells (MNC) infiltration and defused MPO-positive granulocytes were noted in the submucosa and mucosa of the old mice in the cardia region. MNC aggregates between the glands spanned the entire width of the mucosa (**Figures 6** and **7**). Intestinal tissues from uninfected and *L. monocytogenes*-infected mice of both age groups were further measured for immune-biomarkers expression (IFN-γ, IL-17a, IL-23 and IL-10 mRNA) (**Figure 8**). *L. monocytogenes* infection increased *in vivo* inflammatory cytokine mRNA responses in the intestinal tissue from old mice which corelated with histological inflammation observed before.

## Discussion

Mice are resistant to oral *L. monocytogenes* infection mostly due to mismatch in the species-specificity of pathogen interaction with corresponding host cells receptors (20), and also, in some part, due to commensal intestinal microbiota responsible for colonization resistance (18). We employed a murinized *L. monocytogenes* strain which is capable of breaching murine intestinal epithelial layers when mice are intragastrically infected (6, 20). A previous study demonstrated that aging is linked to an altered gut microbiota composition, inflammation, and increased gut permeability (34). Commensal bacteria can be recognized by the innate immune system and that individual species or groups of commensal bacterial species can influence distinct modules of innate and adaptive immune response (35). Therefore, any dysbiosis of commensal due to aging or infection can modulate immune response.

In our current study, we evaluated if changes in pathological and immune-biomarker response in old mice had any positive-correlation with an altered commensal microbiota population. *Listeria* infection caused mild inflammation in young-adult mice, but more inflammation and pathology in older mice. We also observed altered gut microbiota composition in the old mice prior to infection. In fact, such perturbation or dysbiosis can happen as mice age (9, 36) and infection may further alter that dynamic. As shown in **Figures 6** and **7**, infected older mice had higher gastric pathology, with marked infiltration of polymorphonuclear neutrophil (PMN). We also observed relatively low-level inflammation in old mice even before infection. Our metagenomics data showed that aging resulted in significant dysbiotic changes in the fecal microbiota population, for example, decreased diversity and lower relative abundance of specific anti-listerial species of bacteria, like *Clostridiales* and *Lactobacillus*. Reduced abundance of these important commensals may compromise colonization resistance in older mice allowing increased *L. monocytogenes* infection through the oral route, intestine and further disseminating into the systemic sites, including liver (**Supplementary Figure 1**).

We also seen the presence of *Parabacteroides_unclassified*, *Prevotella buccae* and *Blautia_unclassified* bacteria in young-adult mice (**Figure 5A** **)**. Both *Parabacteroides_unclassified* and *Prevotella buccae* which belong to *Porphyromonadaceae* and *Prevotellaceae* family respectively, were increased after infection in young-adult mice. The *Porphyromonadaceae* and *Prevotellaceae* families are commensal and associated with inflammatory response to infection (37, 38). The abundance of *Parabacteroides_unclassified* decreased in infected old mice (**Figure 5C**) and those mice also had increased anti-inflammatory (IL-10) response. On the other hand, *Blautia_unclassified*, which belongs to *Lachnospiraceae* family were significantly decreased in infected young-adult mice as compared to uninfected mice. Murri et al. (39) reported that *Blautia* abundance is associated with a healthy gut microbiome and their abundance can decrease during human liver diseases and in certain cancers, which may be correlated with its significant reduction in infected-young-adult mice. It is important to note that, *Blautia* was not detected in old mice. *Alistipes finegoldii*, a commensal bacterium belonging to *Rikenellacdeae* family are exclusively present in the old mice and believed to be indicative of gastrointestinal health. Its abundance in the gut has shown to be decreased during gastrointestinal inflammation (36). We observed, *Listeria* infection significantly reduced its abundance in old mice (**Figure 5B**) that may correlate with increased gastric inflammation seen in these mice (**Figures 6** and **8**). As shown before *Pseudoflavonifractor capillosus*, *Feacalibacterium* spp. and *Anaerotruncus* sp. G3 (2012) bacteria, all butyrate producers, were identified as highly abundant in infected old mice, and have been reported to be involved in the regulation of inflammation response during human diseases and infection (34, 36). We observed a significant rise in abundance of these bacteria only in the *L. monocytogenes*-infected old mice as compared to *L. monocytogenes*-infected young-adult mice (**Figure 5C**). Both *Feacalibacterium* spp. and *Anaerotruncus* sp. G3 (2012) species belong to *Ruminococcaceae* family bacteria. One report suggested that *Feacalibacterium prausnitzii* A2-165 strain can induce IL-10 production in dendritic cells and modulate T cell response (40). Notably, our study also showed similar increased anti-inflammatory (IL-10) response (**Figure 8C**) in old infected mice which may correlate the increased abundance of members of the *Ruminococcaceae* family bacteria in the feces from old mice (**Figure 3B**).

The *Lachnospiraceae* family are more abundant in infected old mice (**Figure 3B**), and have been reported to be associated with inflammation and obesity (41, 42); this data correlates with our findings that show a higher inflammatory response in infected old mice (**Figures 6**–**8**). Our histology data suggest that *L. monocytogenes*-infected old mice had a significantly heightened level of inflammatory biomarkers which may correlate with increased diversity of the *Lachnospiraceae* family member bacteria. In addition, the family *Lachnospiraceae* has been reported to have a possible anti-inflammatory role (43, 44), but its specific role has yet to be elucidated. Previously in our study, we reported an increased anti-inflammatory response (IL-10 and Treg cells) in the old mice (7). Furthermore, we observed an increased relative abundance of *Firmicutes* bacterium ASF500 species belonging to phylum *Firmicutes* in old mice as compared to young-adult mice. Interestingly, Atarashi et al. (45) showed that the *Firmicutes* bacterium ASF500 can induce pro-inflammatory IL-17 cells which we also observed in our previous study. Notably, in our current study, intestinal IL-17 and IL-23 mRNA response from infected old mice was also high (**Figures 8B, D**).

The common bacteria in the phylum *Firmicutes*, including the *Clostridium* cluster XIVa, take part in predominant role in the fermentation of carbohydrates within the gut (46). The crucial end products of this fermentation in the gut are various short-chain fatty acids (SCFAs) like, acetate, propionate, and butyrate. *Firmicutes* is the principal bacterial phylum, containing over 250 genera, including *Lactobacillus*, and *Clostridium* which can generate several SCFAs, including butyrate. Butyrate serves as the main source of nutrition for cells of the gut epithelium (47, 48). Depletion, or any change of butyrate levels, is associated with inflammation and impairments in the gut barrier integrity (49, 50). *Akkermansia muciniphila*, a mucin degrading bacterium in the gut is only found in the young-adult mice and not detected in old mice. We did not study the cause-and-effect relationship of any these bacteria mentioned, on their role in inflammatory response *per se*, but suggest that these species could be modulating inflammation *via* SCFAs, including butyrate production as reported by other studies (43, 48). Our results suggest that the decreased proinflammatory response (IFN-γ) and increased anti-inflammatory (IL-10) response we observed in our earlier study in old mice may be, in part, due to an increased abundance of *Lachonospiraceae*. In our current study, we observed increased neutrophil infiltration in intestinal tissue and liver in the Lm-infected old mice that could be due to SCFAs, derived from dysbiosis of commensals. It is now well recognized that SCFAs can regulate immune cells. SFCAs, propionate and acetate, derived from commensal bacteria, promote neutrophil chemotaxis (51) and further focused studies are warranted.

### Conclusion

Foodborne *L. monocytogenes* infection is a public health problem, especially in the susceptible populations (elderly, pregnant, and immune-compromised person). We developed a surrogate mice model that mimics human foodborne listeriosis and investigated the role of gut-microbiota correlating with immune-status on the risk of developing listeriosis. We have shown that aging alters gut microbiota composition and may compromise colonization resistance against *L. monocytogenes* infection. In older mice, species-diversity and abundance is reduced. Specifically, beneficial commensal like *Lactobacilus* spp., and taxa belonging to the order *Clostridiales* are reduced or completely absent in older mice. We hypothesize infection with *L. monocytogenes* in older mice may facilitate increased numbers of specific immune-modulating bacteria belonging to the *Lachnospiraceae* and *Ruminococcaceae* families. *L. monocytogenes* infection in mice can cause marked perturbation of the host gut microbiota and a recent report by Rolhion et al. (52) showed that bacteriocin from *L. monocytogenes* can target the commensal *Prevotella copri* and modulate intestinal infection. We measured tissue inflammation response of young-adult and old mice before and after infection and correlated that response to dysbiosis of commensal bacteria due to aging. We did not study the functional relations/response as to why aging or infection altered the gut microbiota composition and how that can affect immune function. Our study is rather limited to establishing a possible correlation between dysbiosis with increased risk of listeriosis under aging condition. Also, it is still unclear whether the dysbiosis is a cause or consequence of inflammation. We propose that aging may cause significant dysbiosis of commensal microbiota in older mice that may compromise their immune balances. In addition, with the loss of beneficial anti-listerial commensal bacteria, increased *L. monocytogenes* colonization in the gut can occur, that may further perturb immune-modulating bacteria that are responsible for plethora of immune activation resulting in increased risk and disease severity.

## Data Availability Statement

The datasets presented in this study can be found in online repositories. The names of the repository/repositories and accession number(s) can be found below: https://www.ncbi.nlm.nih.gov/, PRJNA691798.

## Ethics Statement

The protocol (protocol approval number: BFQ-11-006) was approved by the Food and Drug Administration, Center for Food Safety and Applied Nutrition-Institutional Animal Care and Use Committee (CFSAN-IACUC). All mice were kept at the MOD-1, CFSAN/FDA, AAALAC (American Association for Accreditation of Laboratory Animal Care) accredited animal facility.

## Author Contributions

MA and CT conceived and designed the research study, provided the administrative oversight, and wrote the manuscript. MA, CT, NH, JG and TB performed the experiments, and analyzed the data. All authors contributed to the article and approved the submitted version.

## Funding

This research was supported by the intramural research program for the Center for Food Safety and Applied Nutrition, U.S. Food and Drug Administration.

## Ackowledgments

The authors are thankful to Drs. Wolf-Dieter Schubert and Thomas Wollert, HZI, Germany, for providing the Lmo-*InlA* ^m^ strain.

## Conflict of Interest

Author NH was employed by the company CosmosID.

The remaining authors declare that the research was conducted in the absence of any commercial or financial relationships that could be construed as a potential conflict of interest.

## Supplementary Material

Supplementary Figure 1 Increased systemic dissemination of *Listeria monocytogenes* (Lm) in old mice after oral infection on 7-days post infection. **(A, B)** Viable *L. monocytogenes* colony count in spleen and liver tissues on 7-dpi. **(C)** Increased colonization of *L. monocytogenes* (green, FITC labeled) in liver tissue detected by immuno-staining with FITC-anti-Lm antibody. Data from mean ± SEM from a representative experiment using 4–6 mice. *p < 0.05, **p < 0.01.
