# Supplementary figures and images for: Aging-Induced Dysbiosis of Gut Microbiota as a Risk Factor for Increased Listeria monocytogenes Infection
Source: Front Immunol. 2021 Apr 28;12:672353. doi: 10.3389/fimmu.2021.672353 (PMC8115019; doi:10.3389/fimmu.2021.672353)

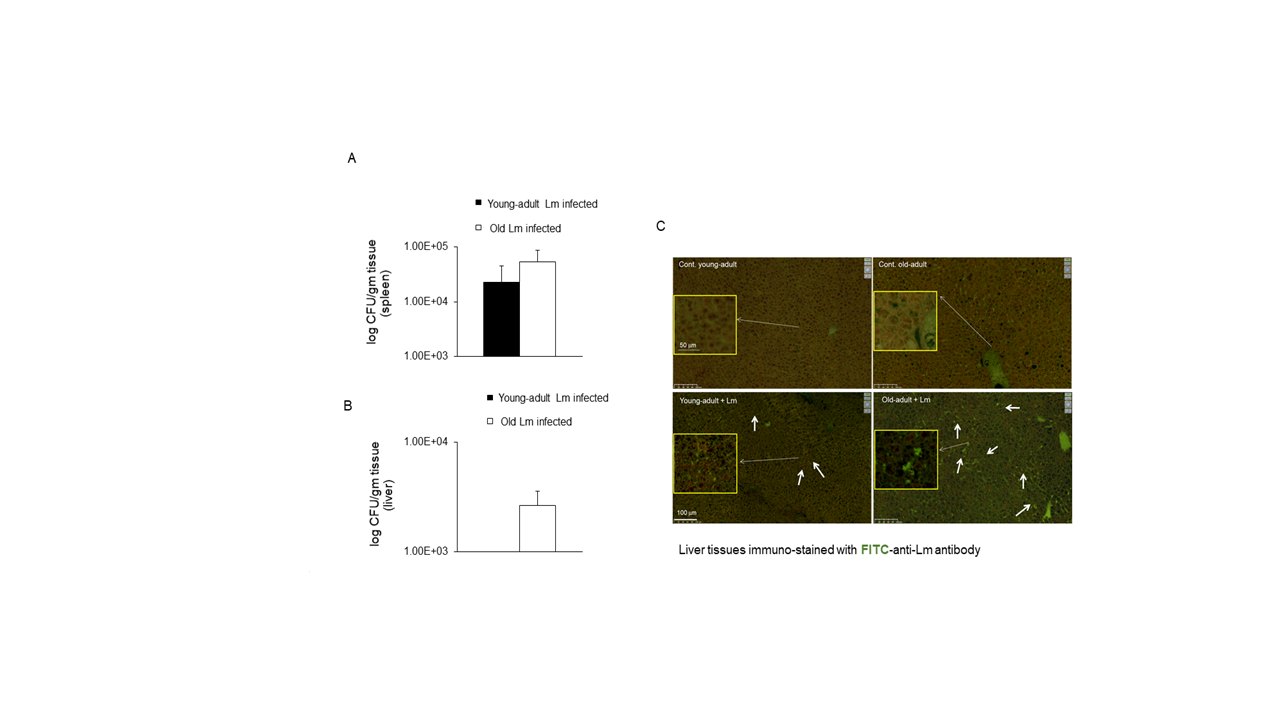

Supplement: Supplementary Figure 1 — Increased systemic dissemination of Listeria monocytogenes (Lm) in old mice after oral infection on 7-days post infection. (A, B) Viable L. monocytogenes colony count in spleen and liver tissues on 7-dpi. (C) Increased colonization of L. monocytogenes (green, FITC labeled) in liver tissue detected by immuno-staining with FITC-anti-Lm antibody. Data from mean ± SEM from a representative experiment using 4–6 mice. *p < 0.05, **p < 0.01. [file Image_1.tif]
